# Nutrient intake and risk of open-angle glaucoma: the Rotterdam Study

**DOI:** 10.1007/s10654-012-9672-z

**Published:** 2012-03-30

**Authors:** Wishal D. Ramdas, Roger C. W. Wolfs, Jessica C. Kiefte-de Jong, Albert Hofman, Paulus T. V. M. de Jong, Johannes R. Vingerling, Nomdo M. Jansonius

**Affiliations:** 1 Department of Epidemiology, Erasmus Medical Center Rotterdam, PO Box 2040, 3000 CA Rotterdam, The Netherlands; 2 Department of Ophthalmology, Erasmus Medical Center Rotterdam, PO Box 2040, 3000 CA Rotterdam, The Netherlands; 3 Department of Pediatrics, Erasmus Medical Center Rotterdam, Rotterdam, The Netherlands; 4 Department of Ophthalmogenetics, The Netherlands Institute for Neuroscience, RNAAS, Amsterdam, The Netherlands; 5 Department of Ophthalmology, Academic Medical Center, Amsterdam, The Netherlands; 6 Department of Ophthalmology, University Medical Center Groningen, University of Groningen, Groningen, The Netherlands

**Keywords:** Glaucoma, Nutrition, Magnesium, Vitamin A, Vitamin B1, Population-based, Dietary intake

## Abstract

Open-angle glaucoma (OAG) is the commonest cause of irreversible blindness worldwide. Apart from an increased intraocular pressure (IOP), oxidative stress and an impaired ocular blood flow are supposed to contribute to OAG. The aim of this study was to determine whether the dietary intake of nutrients that either have anti-oxidative properties (carotenoids, vitamins, and flavonoids) or influence the blood flow (omega fatty acids and magnesium) is associated with incident OAG. We investigated this in a prospective population-based cohort, the Rotterdam Study. A total of 3502 participants aged 55 years and older for whom dietary data at baseline and ophthalmic data at baseline and follow-up were available and who did not have OAG at baseline were included. The ophthalmic examinations comprised measurements of the IOP and perimetry; dietary intake of nutrients was assessed by validated questionnaires and adjusted for energy intake. Cox proportional hazard regression analysis was applied to calculate hazard ratios of associations between the baseline intake of nutrients and incident OAG, adjusted for age, gender, IOP, IOP-lowering treatment, and body mass index. During an average follow-up of 9.7 years, 91 participants (2.6%) developed OAG. The hazard ratio for retinol equivalents (highest versus lowest tertile) was 0.45 (95% confidence interval 0.23–0.90), for vitamin B1 0.50 (0.25–0.98), and for magnesium 2.25 (1.16–4.38). The effects were stronger after the exclusion of participants taking supplements. Hence, a low intake of retinol equivalents and vitamin B1 (in line with hypothesis) and a high intake of magnesium (less unambiguous to interpret) appear to be associated with an increased risk of OAG.

## Introduction

One of the most important sight-threatening eye disorders in ophthalmology is open-angle glaucoma (OAG). This progressive neurodegenerative disease leads to glaucomatous optic neuropathy and eventually, through glaucomatous visual field loss, to loss of sight. Together with age-related macular degeneration it is the most common cause of irreversible blindness.

An increased intraocular pressure (IOP) is the main risk factor for OAG, but IOP-independent mechanisms play a role as well: some people develop OAG without an elevated IOP and in others the deterioration continues despite an apparently sufficient reduction of the IOP. Neurodegeneration and an impaired ocular blood flow are examples of mechanisms supposed to contribute to the IOP-independent deterioration in OAG; oxidative stress is presumed to be part of the neurodegenerative process [1–4]. Apart from its neurodegenerative effect on the optic nerve, oxidative stress has also been suggested to damage the trabecular meshwork, resulting in an increase in the IOP [2]. For these reasons, the effects of nutrients with anti-oxidant activity are of great interest [5], especially because the intake of nutrients is modifiable. It has been shown that differences in intake of anti-oxidants may influence the course of an eye disease (e.g., age-related macular degeneration), even in the western world without apparent malnutrition [6, 7]. Common nutrients with anti-oxidant activity are carotenoids (present in most fruits and vegetables), retinol equivalents, vitamins B, C and E, and polyphenolic flavonoids (present in tea, especially green tea, and coffee).

The second presumed mechanism for an IOP-independent deterioration in OAG was an impaired blood flow. Nutrients of interest here are the omega-3 and omega-6 fatty acids, and magnesium. The fatty acids are well known in the cardiovascular literature because of their effect on blood flow regulation by serving as direct precursors of prostaglandins. It has been suggested that they may be of therapeutic value in OAG [8, 9]. Previous studies suggested that magnesium may play a role in the pathogenesis of OAG by affecting the peripheral circulation [10]. Thus far, the possible role of nutrients in OAG was addressed only in the Nurses’ Health Study (see “Discussion”) [11–13].

The aim of the present study was to explore the influence of nutrients that either have anti-oxidant activity or influence the (ocular) blood flow on the development of OAG. For this purpose, we studied the associations between the intakes of these nutrients and incident OAG in a prospective population-based setting. As there may be effects both directly on the optic nerve and through the IOP, we also studied the associations between these intakes and the IOP.

## Methods

### Participants

The present study was performed within the Rotterdam Study, a prospective population-based cohort study comprising 7,983 residents aged 55 years and older living in Ommoord, a district of Rotterdam, the Netherlands [14]. The baseline examination of the ophthalmic part of the Rotterdam Study included 6,806 participants and took place between 1991 and 1993; follow-up examinations for OAG were performed from 1997 to 1999 and from 2002 to 2006. Participants were included in this study if they (1) participated both at baseline and at least at one follow-up examination, (2) had complete data on OAG and dietary intake, and (3) were not classified as having OAG at baseline. All measurements were conducted after the Medical Ethics Committee of the Erasmus University had approved the study protocol and all participants had given a written informed consent in accordance with the Declaration of Helsinki.

### Ophthalmic examination

The examinations at baseline and follow-up included autorefraction (Topcon RM-A2000; Tokyo Optical Co., Tokyo, Japan), keratometry (Topcon OM-4 Ophthalmometer; Tokyo Optical Co., Tokyo, Japan), measurement of the best corrected visual acuity with ETDRS optotypes, measurement of the IOP (see below), fundus photography of the posterior pole (Topcon TRC-50VT, Tokyo Optical Co., Tokyo, Japan), simultaneous stereoscopic fundus photography of the optic nerve head (Topcon ImageNet System, Topcon TRC-SS2, Tokyo Optical Co., Tokyo, Japan), imaging of the optic nerve head with the Heidelberg Retina Tomograph (Heidelberg Engineering, Dossenheim, Germany) and visual field testing (HFA II 740; Zeiss, Oberkochen, Germany; see below).

The IOP was measured at baseline and at every follow-up round with Goldmann applanation tonometry (Haag-Streit AG, Bern, Switzerland) after applying oxybuprocaine 0.4% eye drops and fluorescein from a paper strip. Three measurements were taken on each eye and the median value of these three measurements was recorded [15]. In the analysis we used the highest IOP of both eyes.

The visual field of each eye was screened using a 52-point supra-threshold test that covered the central visual field with a radius of 24° [16, 17]. Visual field loss was defined as non-response in at least three contiguous test points (or four including the blind spot). In participants with reproducible abnormalities on supra-threshold testing, Goldmann perimetry (Haag-Streit AG, Bern, Switzerland; baseline and first follow-up) or a full-threshold HFA 24-2 test (second follow-up) was performed on both eyes. Details about the classification process were described before [16, 18]. Cases had to have an open anterior chamber angle; a history or signs of secondary glaucoma were not allowed.

Incident OAG was defined as no glaucomatous visual field loss in both eyes at baseline and glaucomatous visual field loss in at least one eye at follow-up [18].

### Assessment of nutrient intake

All participants were interviewed at baseline for food assessment using an extensive semiquantitative food frequency questionnaire at the study center by a trained dietician. The questionnaire was validated and adapted for use in the elderly [19–21]. It contains 170 food items in 13 food groups and questions about dietary habits and supplementation. The dietary intake of nutrients was calculated using The Dutch Food Composition Database (NEVO) from 1993 to 2006. “Energy-adjusted” nutrient intakes were computed as the unstandardized residuals from a (linear) regression model in which total caloric intake served as the independent variable and the absolute nutrient intake as the dependent variable. Because residuals have a mean of zero and thus include negative values, the expected mean nutrient intakes of the study population was added to the residuals as derived from the regression analysis [22]. Next, we divided the energy-adjusted intake into tertiles (low, medium, and high intake). This process was repeated for all nutrients.

Included nutrients were those that have anti-oxidative properties, being carotenoids (α-carotene, ß-carotene, lutein, zeaxanthin, ß-cryptoxanthin and lycopene), vitamins (retinol equivalents, B1, B6, B12, E and C) and flavonoids, and those that influence the blood flow, being omega-3 fatty acids (α-linolenic acid [ALA; C18:3 (n-3) *cis*], eicosapentaenoic acid [EPA; C20:5 (n-3) *cis*], docosahexaenoic acid [DHA; C22:6 (n-3) *cis*]), omega-6 fatty acids (linoleic acid [LA; C18:2 (n-6) *cis*]) and magnesium. Calcium and phosphorus were included because they compete with magnesium absorption.

As there are two sources of dietary vitamin A (active forms and provitamins), we expressed the amounts of vitamin A in retinol equivalents.

Body mass index was calculated as weight in kilograms divided by height in meters squared. Weight and height were measured at the research center.

### Statistical analysis

Differences in baseline characteristics were analyzed with independent *t* tests and Chi-square statistics. Nutrients with *p* < 0.10 in the univariate comparisons (between participants with and without incident OAG) were included in a multivariate analysis. For the latter, we used a Cox proportional hazard model to calculate hazard ratios (HR) with corresponding 95% confidence intervals (CI) to analyze whether participants had a lower or higher risk of incident OAG. Follow-up duration was used as the time variable. Follow-up duration was counted from the baseline visit to the last visit with reliable perimetry. Incident OAG cases were censored at the first visit in which glaucomatous visual field loss was detected. The model was adjusted for age, gender, IOP, IOP-lowering treatment and body mass index, all measured at baseline. Adjustment for IOP was done to analyse whether the effects of the nutrients were independent of IOP. For each nutrient, the lowest tertile (i.e., the participants with the lowest intake) served as the reference group. Tertiles were analyzed as nominal categories, that is, we did not assume beforehand monotonic relationships between nutrient intakes and outcome measure. To evaluate these relationships, linearity was tested by calculating the *p*-trend for the nutrients that showed a significant association with OAG in the final model. We also explored whether there was interaction between age or gender and each of the nutrients from the final model. As a secondary analysis, we repeated the above-mentioned analysis after exclusion of participants who reported to have used any supplements (vitamins or other health pills) at baseline.

To assess the associations between nutrients and IOP, we performed for each nutrient a linear regression analysis (IOP at follow up versus intake). These analyses were adjusted for IOP-lowering treatment at follow-up. Nutrients were analyzed as nominal categories (see above). Nutrients with *p* < 0.10 were included in a multiple linear regression analysis with IOP at follow-up as the dependent variable. This model was adjusted for age, gender, body mass index, and IOP-lowering treatment during follow-up.

We used complete case analysis and considered a *p* value of 0.05 or less statistically significant. All statistical analyses were performed using SPSS version 15.0.0 for Windows (SPSS inc., Chicago, IL, USA; 2006) and R statistical package version 2.11.1 for Mac (http://www.r-project.org).

## Results

A total of 6,806 participants attended the baseline examinations of the ophthalmic part of the Rotterdam Study. From these participants, 6,630 did not have OAG at baseline and 3,939 of 6,630 (59.4%) completed at least one follow-up round (1,279 died before the first follow-up round, 1473 were unable to participate in the first follow-up round and 143 had unreliable data with regard to OAG; of the participants without (reliable) data at the first follow-up round, 204 successfully completed the second follow-up round). Of these 3,939 participants, 437 did not have reliable data of the food frequency questionnaire or supplement use. This resulted in 3,502 eligible participants for the current study.

During a mean follow-up of 9.7 years (range 5.3–13.9 years), 91 (2.6%) developed OAG. Table 1 summarizes the general characteristics of the study population at baseline according to incident OAG status. Participants who developed OAG were significantly older, more often men, and had a lower body mass index than those without incident OAG. Their use of supplements did not differ from that of participants without incident OAG (*p* = 0.86).

**Table 1:** Baseline characteristics and univariate analyses of the study population with and without incident open-angle glaucoma presented as mean ± standard deviation (range) unless stated otherwise

	Incident open-angle glaucoma (N = 91)	No open-angle glaucoma (N = 3411)	*p* value
Age (year)	67.6 ± 7.0	65.1 ± 6.6	<0.001
Gender (N[%] female)	47 (51.6)	2031 (59.5)	0.13
IOP (mmHg)	17.5 ± 4.8	15.0 ± 3.1	<0.001
IOP-lowering treatment (N[%])	14 (15.4)	73 (2.1)	<0.001
Body mass index (kg/m^2^)	25.5 ± 2.9	26.3 ± 3.5	0.03
Supplement users (N[%])	33 (36.3)	1269 (37.2)	0.86
Diabetes mellitus (N[%])	6 (6.6)	126 (3.7)	0.16

*IOP* intraocular pressure

Table 2 shows the descriptive data of the mean daily dietary intake of nutrients for all participants adjusted for total energy intake, with corresponding univariate comparisons between participants with and without incident OAG. At the *p* < 0.10 level, participants with incident OAG had a lower intake of ß-carotene, retinol equivalents, vitamin B1 and vitamin B12, and a higher intake of magnesium and vitamin E compared to participants without incident OAG. No other differences in nutrition were found between participants with and without incident OAG. Table 3 shows the results of the multivariate analysis, both for all participants and after exclusion of participants using supplements. High intakes of retinol equivalents and vitamin B1 were associated with a decreased risk of OAG, whereas a high intake of magnesium was associated with an increased risk. For these nutrients the crude hazard ratios (that is, only adjusted for age) were statistically significant as well (*p* = 0.019, *p* = 0.047, and *p* = 0.014 for high intake of retinol equivalents, vitamin B1 and magnesium, respectively). The *p*-trend for retinol equivalents was 0.018, for vitamin B1 0.042, and for magnesium 0.014. We found no significant interactions between age or gender and the nutrients. The effects were stronger if we excluded participants using supplements.

**Table 2:** Mean dietary intake of the assessed nutrients by tertile (low, medium and high intake), adjusted for total energy intake for each tertile, with corresponding univariate comparisons between participants with and without incident open-angle glaucoma

Nutrient	Low intake		Medium intake		High intake		*p* value*
	Mean ± SD	Range	Mean ± SD	Range	Mean ± SD	Range	
With anti-oxidative properties							
α-carotene (μg/day)	590.72 ± 205.44	(<852.18)	1059.18 ± 119.34	(852.40–1278.48)	1851.95 ± 996.30	(>1278.76)	0.438
ß-carotene (μg/day)	2431.61 ± 613.88	(<3216.03)	3775.53 ± 334.72	(3219.18–4399.33)	5879.44 ± 2609.44	(>4399.61)	0.075
Lutein (μg/day)	1417.24 ± 318.64	(<1842.51)	2164.79 ± 188.46	(1842.72–2504.99)	3235.32 ± 1156.42	(>2506.38)	0.238
Zeaxanthin (μg/day)	72.54 ± 17.16	(<96.48)	117.06 ± 11.85	(96.49–137.88)	175.99 ± 46.14	(>137.89)	0.865
ß-cryptoxanthin (μg/day)	74.77 ± 53.60	(<173.59)	265.80 ± 61.07	(174.27–371.22)	530.05 ± 191.52	(>371.31)	0.809
Retinol equivalents (mg/day)	0.57 ± 0.10	(<0.68)	0.76 ± 0.04	(0.68–0.84)	1.11 ± 0.46	(>0.84)	0.003
Vitamin B1 (mg/day)	0.87 ± 0.11	(<1.01)	1.11 ± 0.06	(1.01–1.22)	1.39 ± 0.15	(>1.22)	0.017
Vitamin B6 (mg/day)	1.31 ± 0.14	(<1.47)	1.59 ± 0.07	(1.47–1.70)	1.92 ± 0.21	(>1.70)	0.988
Vitamin B12 (mg/day)	2.78 ± 0.75	(<3.69)	4.33 ± 0.39	(3.70–5.07)	8.48 ± 6.92	(>5.08)	0.066
Vitamin C (mg/day)	72.07 ± 17.17	(<96.49)	114.99 ± 10.87	(96.50–134.74)	177.97 ± 45.30	(>134.79)	0.956
Vitamin E (mg/day)	8.83 ± 1.99	(<11.38)	13.29 ± 1.13	(11.38–15.33)	19.42 ± 4.04	(>15.35)	0.086
Flavonoids	16.52 ± 4.77	(<23.09)	27.94 ± 2.89	(23.10–33.23)	42.18 ± 8.70	(>33.23)	0.923
Affecting blood flow							
ALA (C18:3 (n-3) *cis*) (g/day)	0.62 ± 0.14	(<0.81)	0.95 ± 0.09	(0.81–1.12)	1.56 ± 0.44	(>1.12)	0.470
EPA (C20:5 (n-3) *cis*) (g/day)	0.00 ± 0.01	(<0.01)	0.03 ± 0.01	(0.01–0.05)	0.11 ± 0.09	(>0.05)	0.729
DHA (C22:6 (n-3) *cis*) (g/day)	0.02 ± 0.01	(<0.04)	0.07 ± 0.02	(0.04–0.10)	0.21 ± 0.14	(>0.11)	0.704
LA (C18:2 (n-6) *cis*) (g/day)	6.37 ± 2.47	(<9.47)	12.04 ± 1.56	(9.48–14.94)	19.87 ± 4.54	(>14.94)	0.410
Calcium (mg/day)	773.27 ± 149.99	(<964.91)	1095.41 ± 76.58	(965.19–1238.44)	1527.75 ± 285.00	(>1238.61)	0.358
Phosphorus (mg/day)	1117.50 ± 144.93	(<1319.64)	1515.03 ± 130.32	(1320.59–1786.33)	2498.47 ± 777.77	(>1787.00)	0.865
Magnesium (mg/day)	252.94 ± 28.63	(<287.87)	309.41 ± 12.38	(287.88–331.51)	369.48 ± 34.67	(>331.52)	0.001

* *p* Values of the univariate analyses between participants with and without incident open-angle glaucoma

*sd* standard deviation, *ALA* α-linolenic acid, *EPA* eicosapentaenoic acid, *DHA* docosahexaenoic acid, *LA* linoleic acid

**Table 3:** Multivariate analysis for nutrition and open-angle glaucoma, for all participants and after exclusion of participants taking supplements

	Hazard ratio (95% CI; *p* value)	Hazard ratio (95% CI; *p* value)*
Age (year)	1.07 (1.04–1.11; <0.001)	1.09 (1.05–1.14; <0.001)
Gender	0.74 (0.48–1.15; 0.178)	0.72 (0.41–1.25; 0.243)
IOP (mmHg)	1.16 (1.10–1.22; <0.001)	1.17 (1.10–1.24; <0.001)
IOP-lowering treatment	3.87 (1.95–7.69; <0.001)	6.21 (2.68–14.41; <0.001)
Body mass index (kg/m^2^)	0.93 (0.87–0.99; 0.026)	0.96 (0.89–1.04; 0.372)
ß-carotene		
1st	1.00 (reference)	1.00 (reference)
2nd	1.62 (0.97–2.71; 0.063)	0.99 (0.52–1.86; 0.966)
3rd	1.08 (0.59–2.00; 0.795)	0.97 (0.47–2.00; 0.930)
Retinol equivalents		
1st	1.00 (reference)	1.00 (reference)
2nd	1.16 (0.72–1.87; 0.543)	1.04 (0.57–1.89; 0.901)
3rd	0.45 (0.23–0.90; 0.023)	0.33 (0.14–0.80; 0.014)
Vitamin B1		
1st	1.00 (reference)	1.00 (reference)
2nd	0.40 (0.21–0.77; 0.006)	0.28 (0.11–0.68; 0.005)
3rd	0.50 (0.25–0.98; 0.044)	0.39 (0.17–0.90; 0.027)
Vitamin B12		
1st	1.00 (reference)	1.00 (reference)
2nd	1.60 (0.97–2.64; 0.066)	1.09 (0.55–2.16; 0.800)
3rd	0.95 (0.51–1.78; 0.882)	1.37 (0.65–2.89; 0.415)
Vitamin E		
1st	1.00 (reference)	1.00 (reference)
2nd	0.69 (0.39–1.22; 0.199)	0.87 (0.44–1.74; 0.696)
3rd	1.34 (0.81–2.22; 0.250)	1.27 (0.66–2.48; 0.476)
Magnesium		
1st	1.00 (reference)	1.00 (reference)
2nd	0.54 (0.27–1.07; 0.076)	0.53 (0.21–1.35; 0.185)
3rd	2.25 (1.16–4.38; 0.016)	3.19 (1.42–7.20; 0.005)

* Participants using supplements were excluded (N = 1302, of which 33 with open-angle glaucoma, leaving 58 open-angle glaucoma cases)

*CI* confidence interval, *IOP* intraocular pressure

As mentioned in the Introduction, factors may affect the optic nerve head in OAG directly or through the IOP. In the latter case, IOP is in the causal pathway. Although the hypotheses suggest IOP-independent mechanisms for the effects of nutrient intake on OAG, we repeated our analyses without adjustment for IOP and also studied the role of IOP separately. The results of our primary analysis (Table 3) did not change after removing IOP from the model, i.e., the effects appeared to be IOP independent. In the multiple linear regression analysis, there were significant associations for a medium intake of ß-cryptoxanthin (beta: -0.35 mmHg; *p* = 0.049) and a medium intake of vitamin B12 (beta: -0.35 mmHg; *p* = 0.039) with IOP. A high intake of these two nutrients, however, was not significantly associated with IOP, precluding the presence of a clear dose–response relationship.

## Discussion

Retinol equivalents and vitamin B1 seem to have a protective effect on OAG, whereas magnesium intake appears to be associated with an increased risk. Since the analyses were adjusted for IOP, these effects should be considered IOP-independent. This is in agreement with the fact that we did not find a significant effect of these nutrients on IOP. None of the other nutrients had an unequivocal effect on IOP as well. The effects of retinol equivalents, vitamin B1 and magnesium on OAG became stronger when excluding participants taking supplements.

Little is known about the possible associations between nutrition and OAG. The Nurses’ Health Study and the Health Professionals Follow-up Study, two large prospective cohorts comprising over 100,000 participants, studied the influence of nutrients with anti-oxidative properties on OAG. They could not find a significant association [11]. Our results on anti-oxidants (with the exception of vitamin A) were in line with their results (no significant effect on OAG). A possible explanation for the non-significant finding of vitamin A in their study might be that their OAG cases were based on self-report. After all, glaucoma is an insidious eye disease and about half of the OAG patients are undetected at a certain moment [17, 23]. This may cause a selection bias in studies based on self-report (or studies performed in a clinical environment). Our population-based approach in which the OAG cases were detected by systematic screening only should be robust with regard to such a selection bias. A striking difference between the Nurses’ Health and Health Professional Follow-up Study and our study is a difference in baseline IOP: 75.5% of the participants in the Nurses’ Health and Health Professional Follow-up Study had an IOP > 22 mmHg compared to only 15.4% in our study. Furthermore, participants of the Nurses’ Health Study and the Health Professional Follow-up Study were younger (30–75 years; mean age ~49 years) compared to those from the current study (>55 years; mean age 65.2 years). Dietary intake, metabolism, and absorption of nutrients are different in the elderly. The effect of vitamin A on the development of OAG may be different in young people developing high-tension glaucoma compared to elderly people developing normal-tension glaucoma. Unfortunately, power limitations inhibited the conduct of interaction analyses between vitamin A, baseline IOP and age. Of interest is that vitamin A and retinoic acid (a metabolite of vitamin A) were shown to have a therapeutic effect in Alzheimer’s disease, like OAG a neurodegenerative disorder [24, 25]. This effect may be due to the anti-amyloidogenic properties of retinoic acid (and vitamin A) [26]. Several studies implicated amyloid-beta deposition in Alzheimer’s disease. It has been shown that amyloid-beta is involved in retinal ganglion cell apoptosis in OAG [27]. In animals, retinoic acid plays a role in optic nerve regeneration after injury [28]. Food sources rich in retinol equivalents are dairy products (milk, cheese, butter) and liver.

Patients with OAG are reported to have a lower thiamine (vitamin B1) level than controls [29]. This supports the current finding of a protective effect of vitamin B1. We showed the protective effect of vitamin B1 on OAG to be independent of IOP. This is in agreement with the association between vitamin B1 deficiency and degeneration of ganglion cells of the brain and spinal cord in animal experiments [30], and between vitamin B1 deficiency and a reduced thickness of the retinal ganglion cell layer in rats [31]. Furthermore, a link between vitamin B1 deficiency and other optic neuropathies is well established [32]. Obviously, most participants with a low intake of vitamin B1 in the current study may not have had a manifest vitamin B1 deficiency. Alcohol consumption could be a confounder here, but alcohol consumption was not associated with either IOP or OAG [33]. Recently, a synthetic derivative of vitamin B1, sulbutiamine, has been shown to have a neuroprotective effect on retinal ganglion cells, probably due to its anti-apoptotic properties [34]. Food sources rich in vitamin B1 are grain products (bread, rice) and potatoes.

Regarding nutrients affecting blood flow, the Nurses’ Health Study and the Health Professionals Follow-up Study showed an increased risk of OAG in participants with a high ratio of omega-3 to omega-6 polyunsaturated fat (relative risk [95% CI]: 1.49 [1.11–2.01]) [12]. However, the latter association was only present in participants with high-tension OAG (OAG with IOP > 21 mmHg; relative risk [95% CI]: 1.68 [1.18–2.40]) and not in participants with normal-tension OAG (OAG with IOP ≤ 21 mmHg; relative risk [95% CI]: 0.82 [0.20–3.47]). The ratio of omega-3 to omega-6, adjusted for total energy intake, was in our study higher in participants with OAG (mean 0.19) compared to participants without OAG (0.12), but this difference did not reach statistical significance (*p* = 0.22). This lack of significance might be explained by power limitations or by the fact that the majority (79%) of our incident OAG cases had an IOP ≤ 21 mmHg and were untreated at baseline. The corresponding small number of cases with an IOP > 21 mmHg or on IOP-lowering treatment at baseline hampers a secondary subgroup analysis.

A review of natural therapies (that is, by means of changing nutrition habits) for ocular disorders highlighted the role of magnesium supplementation in OAG therapy [30]. Magnesium administration improves the peripheral circulation [35] and has been reported to have a beneficial effect on the visual field in patients with OAG with cold-induced vasospasm [10]. However, there are some concerns regarding the latter study. They included only ten glaucoma patients (of which six with OAG) and observed an improvement after a very short follow-up. This suggests a short-term effect on the sensitivity of the visual system rather than a real effect on glaucoma progression. Moreover, the observed change was small and did not reach statistical significance (*p* = 0.09) [36]. As mentioned in the Methods section, magnesium absorption competes with that of phosphorus and calcium; however, we found no significant interaction between these nutrients (data not shown). Furthermore, magnesium is present in many fruits, vegetables, beans and dairy products, and thus, the association of magnesium with OAG might be explained by other nutrients present in magnesium-rich products. Interestingly, the rationale of magnesium substitution is that magnesium has similar effects as calcium channel blockers [10, 37]. A double-blind controlled trial with oral magnesium aspartate HCl supplements, presented an inverse association with systemic blood pressure [38]. The role of calcium channel blockers in OAG, however, is all but clear. Several clinical studies suggested a small protective effect [39–41], but two large epidemiological studies including our study reported a harmful effect. These contradictory findings may be attributed to patient selection [42, 43]. Adjusting for the use of calcium channel blockers at baseline did not alter the harmful effect of magnesium in our study (data not shown). As the influence of calcium channel blockers on OAG is unclear, it is difficult to interpret our findings on magnesium.

The strengths of the present study are its population-based setting and the long follow-up. The study population presumably consumed a healthy diet as the median nutrient intake of the study population was at or above the recommended daily allowance [6]. A possible limitation of our study is that only one food frequency questionnaire was used at baseline. Although this could initiate misclassification (dietary changes may result, amongst others, as part of treatment for chronic illnesses like hypertension, diabetes mellitus and age-related macular degeneration), such misclassification would unlikely result in false-positive findings (none of the illnesses that have dietary measures as part of its treatment are clearly associated with OAG). Another possible limitation is the limited number of participants who developed OAG during follow-up. Almost half of the cohort deceased or did not participate at follow-up. Although a selective non-response is possible, we found no significant differences in dietary intake of nutrients or intake of supplements between participants and non-participants. The only relevant differences in baseline characteristics were age and gender: non-participants were significantly older and more often women [18]. As high age is associated with an increased risk of OAG and a decreased dietary intake, non-participation might have resulted in an underestimation of our findings. No differences in IOP or body mass index were observed between participants and non-participants. As we tested a large number of nutrients, this might have resulted in false-positive findings. To cope at least partially with this, we only included nutrients that were earlier addressed in the literature or for which a clear pathophysiological rationale could be found (see “Introduction” section). If we would apply the very strict Bonferroni correction for multiple comparisons to the univariate analyses as presented in Table 1, a *p* value of 0.003 (0.05/19) would have been considered as statistically significant in the univariate analysis. According to Table 1 magnesium and the retinol equivalents are still significant at this *p* value.

The percentage of participants taking supplements in our study may seem high (36.2–37.2%; Table 1), however, it was very similar to other studies conducted in the early nineteens (the baseline of this study). For example, the National Health and Nutrition Examination Survey reported that in the age range of 60–69 years, 39.2% of men and 51.6% of women used supplements in the United States [44]. A study from Sweden reported supplement use in 22.2% in men and 33.3% in women [45]. Finally, we did not adjust for variables like diabetes mellitus, blood pressure and cholesterol level, as was done in the Nurses’ Health Study and the Health Professionals Follow-up Study [11]. However, in the present study none of these variables showed significant univariate differences at baseline (*p* = 0.158, *p* = 0.976 and *p* = 0.682, respectively; Table 1), and thus they would have been excluded from the final model—if they would have been included initially. The same holds for smoking; previously, we reported no association between smoking and OAG [33]. Although diabetes mellitus was reported not to be associated with OAG in the Rotterdam Study [46], some studies suggest the opposite. Furthermore, magnesium has been associated with diabetes mellitus. For these reasons, we performed an additional analysis by adding diabetes mellitus to the model. This did not change the findings (data not shown).

In conclusion, the current findings suggest that people with a high intake of retinol equivalents or vitamin B1 have an about twofold lower risk of OAG compared to those with a low intake of these nutrients, and people with a high intake of magnesium have an about threefold increase in risk of OAG compared to those with a low intake. These findings might be helpful in the unraveling of the largely unknown pathophysiology of OAG.
